# P-1266. Understanding Factors that Influence Patients’ Decision to Seek Care for Flu-Like Symptoms in Emergency Departments During Seasonal Respiratory Viral Surges

**DOI:** 10.1093/ofid/ofae631.1448

**Published:** 2025-01-29

**Authors:** Allison K Jacobi-Dorbeck, Rose-Alice Duverna, Claire S Wilson, Breana McBryde, Richard E Rothman, Mustapha Saheed, Bhakti Hansoti

**Affiliations:** Johns Hopkins University School of Medicine, Baltimore, Maryland; Johns Hopkins University School of Medicine, Baltimore, Maryland; Johns Hopkins University School of Medicine, Baltimore, Maryland; Johns Hopkins University School of Medicine, Baltimore, Maryland; John Hopkins University, Baltimore, Maryland; Johns Hopkins University School of Medicine, Baltimore, Maryland; Department of Emergency Medicine, Johns Hopkins University, Baltimore MD, Baltimore, Maryland

## Abstract

**Background:**

Emergency departments (EDs) are directly impacted by seasonal surges in rates of respiratory viral infection and transmission with increased rates of ED visits for flu-like symptoms. It remains unclear as to why patients assigned lower acuity (level 3,4 and 5) based on standardized triage scoring systems opt to seek care within the emergency department in lieu of alternative healthcare venues for flu-like symptoms. We conducted an exploratory quality improvement study to develop further understanding of patient-related factors for seeking ED care, including patient perception of illness severity, and how this correlated with final ED disposition.

**Figure:**
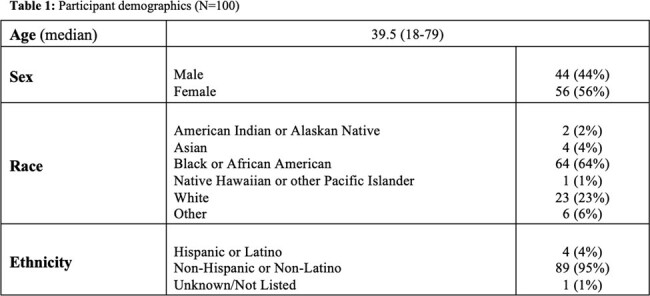

**Methods:**

Patients were recruited from the adult ED using convenience sampling, from 2 major urban hospital centers based on a chief complaint related to flu-like symptoms and a triage score of 3, 4, or 5 (indicating non-emergent clinical status). Patients were asked to complete a structured survey to assess self-perception of current illness severity and reasons for seeking care in the ED. Following survey completion, EMR chart review was performed to collect information about patient’s clinical course, , diagnosis, and disposition.

**Figure:**
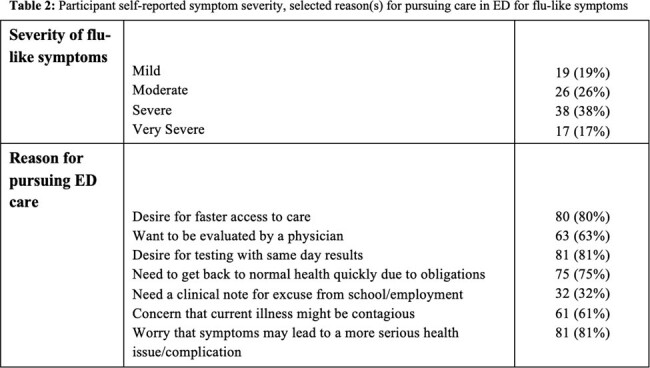

**Results:**

100 subjects were enrolled; over half (55%, *n*=55) graded their symptoms as being ‘severe’ or ‘very severe’ on presentation. Survey results demonstrated that desire for fast access to care (*n* = 80); testing with same-day results (*n* = 81); and worry that symptoms may evolve into a more serious condition (*n* = 81) were the top three most commonly reported reasons for seeking care in the ED. 24/100 participants were admitted and 76 were discharged. Rates of self-reported ‘severe’ or ‘very severe’ illness were nearly identical amongst those who were discharged 43/76 (57%) versus those who admitted 12/24 (50%). .

**Conclusion:**

Amongst lower acuity patients presenting to the ED during flu season, the vast majority sough care in the ED due to a desire to be seen quickly and a concern for having serious illness. We observed surprisingly high rates of hospital admission (25%); patients’ perception of having a more severe illness was similar amongst those discharged (versus admitted) suggesting that patients are not able to reliably predict a need for urgent ED evaluation.

**Disclosures:**

**Richard E. Rothman, MD, PhD**, abbvie: Grant/Research Support|CEPHEID: Advisor/Consultant|CEPHEID: Grant/Research Support|CEPHEID: Honoraria

